# Rhythmic pattern facilitates speech production: An ERP study

**DOI:** 10.1038/s41598-019-49375-8

**Published:** 2019-09-10

**Authors:** Ning Zhang, Qingfang Zhang

**Affiliations:** Department of Psychology, Renmin University of China, Beijing, 100872 China

**Keywords:** Language, Human behaviour

## Abstract

Rhythm affects the speech perception of events unfolding over time. However, it is not clear to what extent the rhythm could affect the processes of sentence speech production. In this event-related potential (ERP) study, we examined whether a particular rhythmic pattern could affect the planning of speech production before articulation. We recorded electrophysiological (EEG) and behavioural (reaction time) data while participants read aloud a target speech in Chinese. Target speeches were sentences or phrases consisting four characters, with regular (e.g., the 2 + 2 pattern; numbers in the brackets represent the number of syllables) or irregular (e.g., 1 + 3) rhythmic patterns, which were preceded by congruent or incongruent musical rhythmic patterns formed by simple pure tones with different temporal intervals. Behavioural and ERP findings indicated a rhythmic priming effect in comparing congruent and incongruent conditions in the regular target speeches, but not in the irregular ones. An early component (N100) that was elicited in response to target speeches that were rhythmically mismatched to primes was linked to the detection of hierarchical linguistic units, which did not conform to expectations. A later negative component (N400) was thought to reflect the violation of expectation on rhythmic pattern in speech production. These findings suggest that rhythmic pattern constrains grammatical and prosodic encoding during speech production, and support the hypothesis that speakers form a grammatical or a prosodic abstract frame before articulation.

## Introduction

Spoken sentence production is the process of generating a series of words in the correct sequence through grammatical and prosodic encoding. There are two core components of the integration process, which was developed from the analysis of errors in natural speech^1,2^. The first part occurs at the functional level, where the meaning of words is integrated into an abstract syntactic structure. The second part occurs at the positional level, where the phonological representations of words are inserted into a planning frame that fixes their locations in a string. This representation, which is formed by combining phonological words and a planning frame, controls the prosodic representation of the sentence’s phonetic forms^3^. In the field of psycholinguistics, numerous studies have investigated the effects of syntactic structure repetition (i.e., the syntactic priming effect) and offered interpretations of how syntactic repetition influences sentence production^3–5^. However, little is known about how prosody affects the planning process of spoken sentence production.

Prosody refers to the properties of an acoustic signal that are irrelevant to the lexical words in the sentence, e.g., stress, intonation, pitch, rhythm, and speech rate^6,7^. In the present study, we only examined one aspect of prosody, speech rhythm, which refers to the temporal organization of acoustic events that unfold over time^8–11^. Researchers define “rhythm” as the temporal structure that underlies the perception and production of utterance^12^. More specifically, we were interested in whether the planning process of speech production could be primed via a simple pure tone pattern in Chinese speech production.

Feng^13^ proposed a model that the interaction between syntax and prosody is bi-directional. Critically, prosody not only constrains syntactic structure, but also activates syntactic operations in Chinese^14,15^. However, a number of studies have demonstrated the interaction among syntax, semantics, and phonology in process of language comprehension^16,17^. Whether this conclusion can be extended to language production is not known.

### Priming effect in speech production and perception

In the literature, a priming task has been discussed as a method for investigating representations and encoding of different linguistic levels in language comprehension and production. For example, studies on syntactic priming have found that speakers prefer to use recently processed syntactic structures when describing a scenario or an event^3,18^. The repetition effect in these studies was found to reflect either an adaptation of the language production system via implicit learning^4^, or the product of coordination between the speakers in conversation^19^. With the priming paradigm, we aim to investigate whether the rhythmic pattern affects the planning of speech production before articulation.

Recently, a few studies have investigated the prosodic priming effect in speech perception and production using behavioural and electrophysiological measures. At the behavioural level, the studies suggested that different aspects of prosody may have different sensitivities to priming in speech production^7,20–23^. In Jungers *et al*.’s study^21^, listeners heard a fast or slow prime sentence followed by a written target sentence matched for length, lexical stress pattern, and syntactic structure. Their main finding was that the speech rates in reading target sentences were affected by the prime rates, which provided evidence of prosodic persistence in speech production. This finding was replicated in a picture description task^20^ and a reading aloud task^22^. Similarly, the rhythmic priming effect was also observed in a speech reading study. Gould *et al*.^13^ manipulated the syllabic stress locations such that they were either congruent or incongruent between primes (a sequence of simple tones) and targets (words). The results showed that reading latencies were faster in congruent syllabic stress patterns than in the incongruent ones, which hinted at an abstract representation of speech rhythm.

By contrast, other studies suggested that certain aspects of prosody, such as intonational phrase boundary and pitch accenting, cannot be primed in speech production^7,22^. Tooley *et al*. manipulated the presence of a boundary at two syntactic locations in auditorily presented sentences (primes). The participants were asked to read the prime sentences first and then covertly or overtly read a visually presented target sentence. Speech analysis showed that the speakers produced pauses at the primed locations in the primes; however, these effects did not carry over to the target sentences. They also manipulated the pitch of the accenting priming condition in a similar paradigm, and failed to identify the priming effect again. These findings suggested that some aspects of prosody, like the speaking rate, may be more amenable to priming than other aspects of prosody, such as phrase boundaries and pitch accenting.

For speech perception process, Cason and Schön^8^ investigated whether rhythmic priming could enhance phonological processing. Participants heard a simple rhythm prime followed by a French pseudoword that had a matching or mismatching prosodic structure with the prime, and were asked to detect a specific phoneme in the targets. They found that when the target phoneme was presented on-beat with the rhythmic stress, the detection latencies were faster compared to those when the target phoneme was off-beat. Cason *et al*.^24,25^ further demonstrated through auditory-motor training that musical rhythm enhanced the rhythm priming effect. These findings suggested that speakers are sensitive to the temporal changes of rhythmic patterns across music and speech, which may share similar representations.

To explain the rhythmic priming effect, Jones *et al*. proposed a *dynamic attending theory* that assumed that the internal neuronal oscillation entrain was synchronised with external auditory events^26–28^. Specifically, the theory assumed that a person allocated more attentional resources to the time points at which an event occurred (see Pitt & Samuel^29^ for a similar hypothesis in speech perception). This assumption was confirmed by other studies that found that detection performance was better at attended time points than at unattended ones^30^. According to this hypothesis, rhythm primes induce temporal expectation and then facilitate target generation in speech production.

### Electrophysiological study of prosodic priming effect

It is well known that the event-related potentials (ERPs) technique can provide high temporal resolution measures. Cason and Schön^8^ recorded EEG while participants completed a phoneme detection task. Interestingly, they found a larger N100 amplitude for the metrical mismatches than for the matches, which might have reflected a violation of rhythmical expectations. They also found that the target off-beat condition yielded a more positive ERP component and a longer latency than did the on-beat condition in the time window of 250–500 ms (P300). Cason and Schön^8^ found that the difference in the P300 was related to beat expectation in phonological processing. These findings were in line with the assumption of the dynamic attending theory.

Furthermore, a few studies have suggested that the neural oscillation phase reflects the processing of the temporal expectation of metrical structure. In a rhythm perception study, Snyder and Large^31^ reported that gamma-band (20–60 Hz) activity was related to the processing of the rhythmic structure of tone sequences. Wilsch *et al*.^32^ performed an auditory-delayed matching-to-sample task with two syllables. They found that increasing the onset of the first syllable led to increased slow-delta (0.6–0.9 Hz) phase coherence during encoding, and individuals with a higher slow-delta coherence presented decreased alpha power (8–13 Hz) in subsequent memory retention. Arnal, Doelling, and Poeppel found that the coupled delta (1–3 Hz) and beta (18–22 Hz) oscillations temporally aligned with the upcoming targets and bias decisions towards correct responses^33^. These findings, while far from conclusive, suggested that abstract temporal expectations can enhance neural encoding precision via the alignment of neural oscillatory phase.

### The present study

In the literature, Cason *et al*. used musical rhythms as primes and the effect they uncovered was cross-domain, whereas other speech production studies used speech as primes and targets^7,15–18^. Comparisons between the studies suggested that a musical-like prime with the ability to induce temporal expectation may also be used to enhance the planning processes in speech production.

The rhythm is the temporally regular or irregular structure that underlies the perception and production of utterances^12^. Most prior studies used stress-timed languages, such as English or German, which allowed predictions about the position of stressed syllables in a sentence context based on the perceived rhythmic of the preceding sentence context. The rhythmic pattern in Chinese is the combination of words with different numbers of syllables^17^. Most words in Chinese are monomorphemic, monosyllabic words or disyllabic compounds^34^. In written form, Chinese words could be a single or two, or more than two characters, with each character corresponding to a syllable and a morpheme. By contrast, in spoken form, Chinese is a syllable-timed language, which each syllable taking roughly equal time in utterance. Generally, Chinese speakers have a tendency to speak two syllables together in utterances, which is called disyllabification^35^. For a noun phrase or a sentence comprising of four characters, one usually segments the former two characters and the latter two ones into two separate disyllabic words. There is a longer constant interval between two disyllabic words, and a shorter constant interval between two characters (syllables) (see the Materials section). With regard to the prosodic information, a rhythmic pattern [2 + 2] (numbers in brackets are the numbers of syllables) is regular, while a rhythmic pattern [1 + 3] or [3 + 1] is irregular^35^. Note that rhythmic expectation in the current study was not achieved through the stressed and unstressed syllables, but rather through temporal regularity. We assumed that the rhythmic pattern in speech perception could provide a context that led to better predictions and facilitated improved processing in speech production.

For the current study, we used a sequence of simple pure tones with different temporal intervals to form musical-like rhythmic patterns as primes. The rhythmic pattern of the target speech was either congruent or incongruent with the rhythmic pattern of the prime. Participants were asked to read target speeches aloud, and EEGs were recorded concurrently. We hypothesised that a musical-like rhythmic pattern could induce expectations about the target speeches. For reading latencies, we expected to observe a facilitation effect in the congruent prime and target rhythmic pattern when compared with their incongruent equivalents. For ERP data, we expected to observe a difference when comparing the congruent with the incongruent condition, which could occur at an early time window (around 100 ms) and/or at a later time window (around 300–500 ms) before articulation.

## Methods

### Participants

Thirty native speakers of Mandarin Chinese (16 females with a mean age of 20.4 years) from the Renmin University of China participated in the study. All of the participants were neurologically healthy, right-handed, and had normal or corrected-to-normal vision. They were paid for their participation. Informed and written consent was obtained from the participants before the experiment. The Administrative Committee of Psychological Research at Renmin University of China approved this study, and the experiment was performed in accordance with the approved guidelines.

### Stimuli

**Figure 1:**
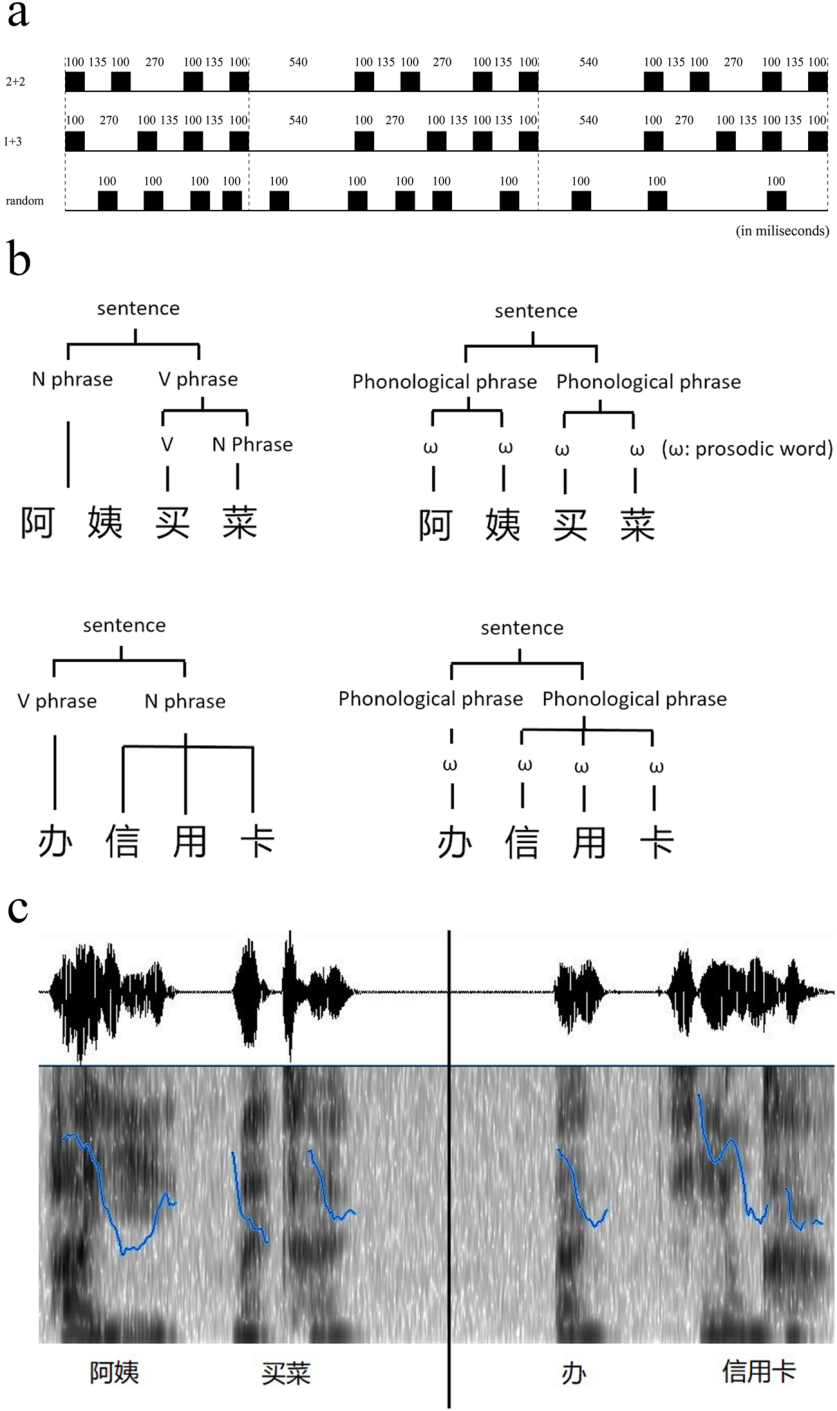
(**a**) Samples of rhythmic patterns for primes (2 + 2: primes with longer interval between the second and the third tone; 1 + 3: primes with longer interval between the first and the second tone). (**b**) Illustration of hierarchical structures of syntax (left) and prosody (right) for a four-word sentence or phrase. (**c**) Illustration of segmentation of target speech patterns of 2 + 2 (left) and 1 + 3 (right) in articulation (Blue lines show the pitch contour of the target speech).

The experimental trials included a prime that was presented auditorily followed by a phrase (or a sentence) that was presented visually. Primes were sequences of four of the same pure tones with a frequency of 900 Hz, and they were created in Adobe Audition CS6. Each prime audio sample consisted of a four-tone rhythmic pattern, which was repeated three times. There were three types of rhythmic patterns used as primes (see Fig. 1a). For the first type, each tone lasted for 100 ms. The interval was 135 ms between the first offsets and the second onsets, and between the third offsets and the fourth onsets, whereas the interval was 270 ms between the second offsets and the third onsets. We named this type of pattern the “2 + 2” where “2” represented two of the same pure tones with a short interval (135 ms), and “+” represented a long interval (270 ms) between two tones. For the second type, each tone lasted 100 ms. The interval was 270 ms between the first offsets and the second onsets, whereas the interval was 135 ms between second offsets and the third onsets, and between the third offsets and the fourth onsets, which resulted in a “1 + 3” rhythmic pattern. The total duration for these two patterns was 940 ms, and each pattern was repeated three times. The interval between each pattern repetition was 540 ms, resulting in a total time of primes for 3.9 s. For the third type, each tone lasted 100 ms, and the interval for the former one’s offset and the latter one’s onset was random, lasting 3.9 seconds in total.

Targets were defined as phrases or sentences that consisted of four Chinese characters. Participants were asked to read targets aloud during the experiment. There were two rhythmic patterns for target speeches (see Fig. 1b for hierarchical linguistic patterns of target speeches at the syntactic and prosodic levels). A character in written form corresponds to a syllable in spoken form in Chinese. One involved a long interval between the second syllable offsets and the third syllable onsets, and the other involved a short interval between the first syllable offsets and the second syllable onsets, which was also the case between the third syllable offsets and the fourth syllable onsets. For example, in the phrase 阿姨买菜 (“the aunt buys vegetables”) the speakers usually segmented the former two characters and the latter two ones into two separate words, i.e., 阿姨 (“the aunt”) and 买菜 (“buys vegetables”). Correspondingly, there was a long interval between two prosodic words, and a relatively short interval between two syllables within a word in articulation, resulting in a 2 + 2 speech pattern. Here, “2” represented the number of syllables, and “+” represented a long interval between two prosodic words. The other involved two words that were separated between the first and the second character within a four-character phrase. For example, the phrase 办信用卡 (“apply for a credit card”) was usually segmented into two words: 办 (“apply for”) and 信用卡 (“a credit card”). Correspondingly, there was a long interval between two words and a relatively short interval among the latter three syllables within a word in articulation, resulting in a 1 + 3 speech pattern. Here, “1” represented one syllable, “3” represented three syllables, and “+” represented a larger interval between two prosodic words (see Fig. 1c). A total of 120 target speeches were composed: half of them were of the 2 + 2 speech pattern, and the other half were of the 1 + 3 speech pattern.

Before the experiment, 25 subjects (none of them participated in the formal experiment) rated the familiarity of target phrases or sentences on a five-point scale, where a value of 1 indicated that a person was very unfamiliar with the target stimulus, and a value of 5 indicated that a person was very familiar with the target stimulus. The average rating scores for both the 2 + 2 and 1 + 3 speech conditions were 4.30 (SD = 0.09) and 4.25 (SD = 0.14), respectively, and they did not significantly differ from each other, *t* (118) = 1.179, *p* = 0.241. This indicated that the two conditions were well matched in familiarity. In addition, there was no significant difference between the stroke numbers between the two conditions (*t* (118) = 1.51, *p *= 0.124), which indicated that the two conditions matched well in terms of visual complexity.

### Design

This experimental design factorially crossed prime rhythm (2 + 2, 1 + 3, and random) and target rhythm (2 + 2 and 1 + 3) variables. Prime rhythm was the within-participants and between-item variables, and target rhythm was the within-participants and between-items variables. Sixty target speeches with 2 + 2 pattern were divided into three sets; each set was paired with prime rhythms of 2 + 2, 1 + 3, and the random, respectively. The identical pairing method was applied to the other 60 target speeches with 1 + 2 pattern. Therefore, there were 6 sets in total, with 20 pairs in each set. Within an experimental block, each participant was presented with 120 trials in total, including 6 different conditions (sets). Additionally, 10 fillers consisting of two- or three-character words were also included within a block. The order of trails within a block was pseudo-randomized in order to prevent the same condition presented more than three times consecutively. A new sequence was generated for each participant and each block. In order to obtain enough trials for the ERP average waveform, each block was repeated three times. It should be noted that a design in which each target is presented and named multiple times is quite common in research on spoken word production, and it is generally considered to be unproblematic.

### Apparatus

The experiment was conducted using E-Prime Professional Software (Version 2.0) in a quiet, electrically and acoustically shielded room. Participants were tested 70 cm from a 19-inch LED computer screen. Prime audios were presented through PHILIPS (SHM7410) headphones. Naming latencies were recorded from the target presentation using a voice-key connected to the computer via a PST Serial Response Box. The articulation responses were recorded by the sound capture toolbox of the E-Prime software through the microphone of the PHILIPS (SHM7410) headphones. The EEG was recorded using AC amplifiers via the Neuroscan 4.5 system (Neuroscan SynAmps).

### Procedure

Participants first completed the Montreal Battery of Evaluation of Amusia (MBEA)^36^ and the Beat Alignment Test (BAT)^37^. Using the two measures, we excluded one participant who had a musical or rhythm perception disorder. Twenty-nine participants without a music disorder and/or temporal perception disorder were entered into the subsequent experimental procedure.

Participants were tested individually. Each experimental trial involved the following sequence. A fixation cross (+) was presented in the middle of the screen for 500 ms, followed by a blank screen for 500 ms. Then, an image of the speaker was presented in the middle of the screen, and the audio of the prime was presented through headphones simultaneously. Participants were required to watch the image of the speaker while listening to the audio. After that, a fixation cross (+) was presented in the middle of the screen for 200 ms, followed by a blank screen for 300 ms. Then, the target phrases or sentences were presented in the middle of the screen. Participants were required to read aloud the phrase or sentence as fast and accurately as possible. If there was no response to the target beyond 10 s, the target would disappear, and then the next trial would begin. The whole procedure of the EEG experiment lasted about 2.5 h.

### EEG recordings and analysis

EEG signals were recorded with 64 electrodes secured in an elastic cap according to the extended 10–20 system. The left mastoid was used as a reference. Two electrodes, each placed on the right and left external canthus, were used to record the horizontal EOG (HEOG). The vertical EOG (VEOG) was recorded with the other two electrodes placed above and below the left eye. All of the electrode impedances were kept below 5 kΩ. A bandpass from 0.05–70 Hz was applied to amplify the EEG signals. The sample rate was set as 500 Hz.

The EEGLAB toolbox, based on MATLAB, was used for the following procedure of preprocessing the EEG signals. The signals were filtered with a high-pass cut-off point of 0.1 Hz and a low-pass cut-off point of 30 Hz. We then ran an independent component analysis on the segmented data (−0.8 s to 1.5 s relative to the sentence onset) to remove the components with artefacts, re-referenced the data to left mastoid, segmented it into epochs of 1000 ms (−200 ms to 800 ms relative to the target onset, with a 200-ms pre-stimulus baseline), and applied baseline correction. We rejected epochs with the criteria of −100 μv to 100 μv. Epochs with latencies out of the range of 500 ms to 992 ms, or with missing/wrong responses, were also deleted. Manual artefact rejection was then applied on the remaining epochs.

## Results

### Behavioural data

**Figure 2:**
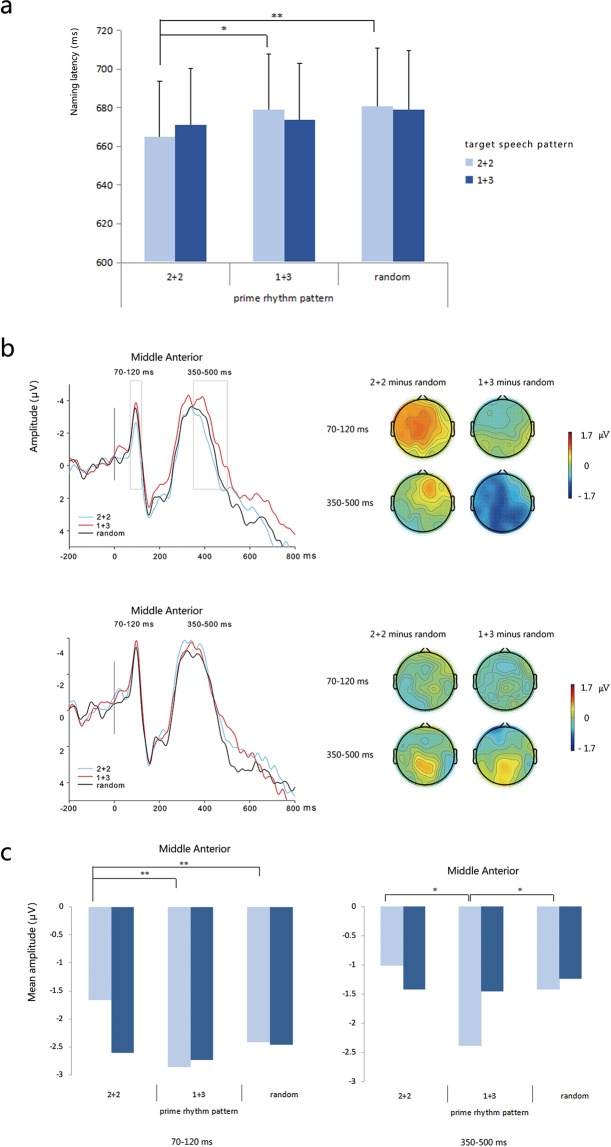
(**a**) Mean latencies of reading target phrase/sentence aloud by prime rhythm pattern and target speech pattern. (**b**) Grand-average ERP waveforms at the area of middle anterior for different prime rhythm patterns in target speech patterns 2 + 2 (top) and 1 + 3 (bottom), respectively. The map (top) shows the distribution of the effect (2 + 2 prime pattern minus random prime pattern and 1 + 3 prime pattern minus random prime pattern) in 2 + 2 target speech pattern in the time windows of 70–120 ms and 350–500 ms, respectively. The map (bottom) shows the distribution of the effect (2 + 2 prime pattern minus random prime pattern and 1 + 3 prime pattern minus random prime pattern) in 1 + 3 target speech pattern in the time windows of 70–120 ms and 350–500 ms, respectively. (**c**) Mean amplitudes of different prime rhythm patterns in target speech patterns 2 + 2 and 1 + 3 at the area of middle anterior, in the time windows of 70–120 ms (left) and 350–500 ms (right).

Trials with incorrect responses (less than 1%) and with latencies shorter than 200 ms or longer than 1000 ms (9.06%) were excluded from the behavioural analyses. The remaining data were used in the subsequent statistical analyses. Figure 2a presents the mean latencies of reading target phrases/sentences aloud through the prime rhythm and target speech patterns.

ANOVAs were performed on the reading latency means with the prime rhythm pattern as the within-participants and between-items variables, and the target rhythm pattern as the within-participants and between-items variables. The effect of the prime rhythm pattern was significant in the participant analysis but not in the item analysis. As *F*_1_ (2, 56) = 3.854, *p* = 0.027, *η*_*p*_^2^ = 0.121; *F*_2_ (2, 114) = 2.117, *p* = 0.125, *η*_*p*_^2^ = 0.036, the effect of the target rhythm pattern was not significant in the participant and item analysis, and *F*_1_ (1, 28) = 0.002, *p* = 0.966, *η*_*p*_^2^ < 0.001; *F*_2_ (1, 114) = 0.016, *p* = 0.900, *η*_*p*_^2^ < 0.001. The interaction between the prime rhythm pattern and target rhythm pattern was not significant in the participant and item analysis, as *F*_1_ (2, 56) = 1.843, *p* = . = 0.168, *η*_*p*_^2^ = 0.062; *F*_2_ (2, 114) = 0.562, *p* = 0.572, *η*_*p*_^2^ = 0.010.

We were also interested in the comparisons between the prime conditions for each target rhythm pattern (regular vs. irregular). Also, researchers suggest that it is appropriate to perform the individual comparisons without depending on a significant omnibus *F* test^38^. Thus, we conducted multiple comparisons between the three prime types using FDR corrections in the 2 + 2 and 1 + 3 target rhythm types. For the targets with the 2 + 2 rhythmic pattern, the difference between the 2 + 2 and random primes was significant (*F*_1_ (1, 28) = 14.866, *p* = 0.0031, *η*_*p*_^2^ = 0.347; *F*_2_ (1, 38) = 6.018, *p* = 0.057, *η*_*p*_^2^ = 0.137), and so was the difference between the 2 + 2 and 1 + 3 primes (*F*_1_ (1, 28) = 5.592, *p* = 0.038, *η*_*p*_^2^ = 0.166; *F*_2_ (1, 38) = 3.440, *p* = 0.107, *η*_*p*_^2^ = 0.083). By contrast, the difference between the 1 + 3 and random primes was not significant (*F*_1_ (1, 28) = 0.250, *p* = 0.621, *η*_*p*_^2^ = 0.009; *F*_2_ (1, 38) = 0.095, *p* = 0.760, *η*_*p*_^2^ = 0.002). For the targets with the 1 + 3 pattern, the difference between any two primes was not significant; all *F*_1_ (1, 28) ≤ 0.126; all ps ≥ 725; all *η*_*p*_^2^ ≤ 034; all *F*_2_ (1, 38) ≤ 613; all *ps *≥ 0.770; and all *η*_*p*_^2^ ≤ 016.

Error rates were considered too low to allow for a meaningful statistical analysis.

### ERP data

Prior to offline averaging, all of the single-trial waves were screened for eye movement, electrode drifting, amplifier blocking, and electromyogram (EMG) artefacts. Trials in which speakers produced incorrect responses and those with onset latencies faster than 500 ms and slower than 992 ms (2SD deviation from the average mean) were excluded from the EEG analysis. The criterion of excluding latencies faster than 500 ms was applied to avoid contamination from artefacts associated with articulation. Data from three participants were excluded due to large EEG artifacts. The remaining epochs (79% of all trials) were baselined using a pre-stimulus baseline and then averaged by condition.

Mean amplitudes were first calculated for each participant and each condition in each time window of 10 ms. Once there were three or more successive time windows obtaining significant differences among the conditions on relatively nearby electrodes, they were merged into a larger time window. Using this procedure, the mean amplitude in the time windows of 70–120 ms and 350–500 ms were chosen for the subsequent statistical analysis. To provide a comprehensive scalp distribution of ERP effects, statistical analysis was conducted on nine ROIs: left-anterior (F3, F5, FC3), middle-anterior (Fz), right-anterior (F4, F6, FC4), left-central (C3, C5, CP3), middle-central (Cz), right-central (C4, C6, CP4), left-posterior (P3, P5, PO3), middle-posterior (Pz), and right-posterior (P4, P6, PO4). An ANOVA was conducted separately for each time window on the ERP amplitude means with the factors the prime rhythm pattern (2 + 2, 1 + 3, and random), the target rhythm pattern (2 + 2, 1 + 3), and the ROIs. The Greenhouse-Geisser correction was applied when appropriate.

In the time window of 70–120 ms, the effect of the prime rhythm pattern was significant (*F* (2, 50) = 4.422, *p* = 0.017, *η*_*p*_^2^ = 0.150), but the effect of the target rhythm pattern was not significant (*F* (1, 25) = 1.317, *p* = 0.262, *η*_*p*_^2^ = 0.050). Crucially, the interaction between the prime rhythm pattern and target rhythm pattern was significant (*F* (2, 50) = 3.976, *p* = 0.025, *η*_*p*_^2^ = 0.137), the effect of the ROI was significant (*F* (8, 200) = 17.976, *p *< 0.001. *η*_*p*_^2^ = 0.418), the interaction between prime rhythm pattern and ROI was not significant (*F* (16, 400) = 0.881, *p* = 0.592, *η*_*p*_^2^ = 0.034), the interaction between target rhythm pattern and ROI was marginally significant (*F* (8, 200) = 1.783, *p* = 0.082, *η*_*p*_^2^ = 0.067), and the interaction among the prime rhythm pattern, target rhythm pattern, and ROI was not significant (*F* (16, 400) = 1.000, *p* = 0.455, *η*_*p*_^2^ = 0.038).

Furthermore, we conducted multiple comparisons between the three prime types using the FRD corrections in the 2 + 2 and 1 + 3 target rhythm types. For the targets with the 2 + 2 pattern, the difference between the 2 + 2 and random prime pattern was significant (*F* (1, 25) = 10.402, *p* = 0.0045, *η*_*p*_^2^ = 0.294), as was the difference between the 2 + 2 and 1 + 3 prime conditions (*F* (1, 25) = 13.993, *p* = 0.003, *η*_*p*_^2^ = 0.359). However, the difference between the 1 + 3 and random prime pattern was not significant (*F* (1, 25) = 1.061, *p* = 0.313, *η*_*p*_^2^ = 0.041). For the 1 + 3 target rhythm, there was no significant difference for any two of conditions; all *Fs* (1, 25) ≤ 0.621; all *ps *≥ 0.438; and all *η*_*p*_^2^ ≤ 0.024.

In the time window of 350–500 ms, the effect of the prime rhythm pattern was not significant (*F* (2, 50) = 2.162, *p* = 0.126, *η*_*p*_^2^ = 0.080), the effect of the target rhythm pattern was not significant (*F* (1, 25) = 1.901, *p* = 0.180, *η*_*p*_^2^ = 0.071), the interaction between the prime rhythm pattern and target rhythm pattern was not significant (*F* (2, 50) = 2.301, *p* = 0.111, *η*_*p*_^2^ = 0.084), the effect of the ROI was significant (*F* (8, 200) = 9.156, *p *< 0.001, *η*_*p*_^2^ = 0.268), the interaction between the prime rhythm pattern and ROI was not significant (*F* (16, 400) = 0.991, *p* = 0.466, *η*_*p*_^2^ = 0.038), the interaction between the target rhythm pattern and ROI was not significant (*F* (8, 200) = 1.004, *p* = 0.434, *η*_*p*_^2^ = 0.039), and the interaction between the prime rhythm pattern, target rhythm pattern, and ROI was not significant (*F* (16, 400) = 1.470, *p* = 0.107, *η*_*p*_^2^ = 0.056).

Furthermore, a similar statistical method was applied for multiple comparisons. For the targets with the 2 + 2 pattern, the difference between the 2 + 2 and random prime condition was not significant (*F* (1, 25) = 0.0165, *p* = 0.919, *η*_*p*_^2^ < 0.001); however, the difference between the 1 + 3 and random prime conditions was significant (*F* (1, 25) = 5.701, *p* = 0.025, *η*_*p*_^2^ = 0.186), and so was the difference between the 2 + 2 and 1 + 3 prime conditions (*F* (1, 25) = 21.551, *p* = 0.0165, *η*_*p*_^2^ = 0.236. For the 1 + 3 target rhythm, there was no significant difference for any two of conditions; all *Fs* (1, 25) ≤ 0.079; all *ps* ≥ 0.781; and all *η*_*p*_^2^ ≤ 0.003.

For the 2 + 2 target rhythm, the waveform in the 1 + 3 prime rhythm (incongruent) was more negative than the one in the 2 + 2 rhythm (concongruent condition) in both time windows (70–120 ms and 350–500 ms). By contrast, for the 1 + 3 target rhythm, the amplitude of the waveform in the 2 + 2 prime rhythm was comparable with the one in the 1 + 3 prime rhythm (see Fig. 2).

## Discussion

Our study aimed to investigate the rhythmic priming effect in speech production. A musical-like rhythmic prime was used to elicit expectations about the whole rhythmic pattern of the target speech. Behavioural results showed a rhythmic priming effect, which presented with faster reading latencies in the congruent prime and target pattern conditions than in the incongruent conditions, but only for the 2 + 2 speech rhythmic pattern, and not for the 1 + 3 speech rhythmic pattern. Critically, ERP results showed an interaction between the prime rhythm and target rhythm in the two time windows (70–120 ms and 350–500 ms). Specifically, we found a rhythmic priming effect for the 2 + 2 target rhythm but not for 1 + 3 target rhythm. Behavioural and electrophysiological results consistently indicated that speakers created an abstract frame in speech production before articulation.

A rhythmic priming effect at the behavioural level indicated that a rhythmic pattern was amenable to priming in speech production. This rhythmic pattern was formed by manipulating the temporal structure among syllables, which suggested that speakers are sensitive to temporal structure in target speech production. This was consistent with previous studies^7,15–17^ in which the speaking rate priming effect was been observed. Speaking rate was manipulated by changing the presentation time of each word in target speeches, which belonged to a type of temporal structure. After the prime’s presentation, speakers created an abstract plan for when to produce a word or a syllable in reading a phrase or a sentence aloud. The model proposed by Tooley *et al*.^7^ suggested that prosodic production is the result of interaction between different levels of representation (i.e., syntax, semantics, and discourse). We hypothesised that there are two possible loci for the rhythmic priming effect: grammatical and prosodic levels in speech production. The underlying mechanisms of the rhythmic priming effect are discussed below in combination with electrophysiological results.

In further elucidating the behavioural findings, associated ERPs provided invaluable additional information based on fine-grained temporal resolution. Across all of the conditions, classic exogeneous P1/N1/P2 ERP components were found within a time window of 0–250 ms. A larger N100 component in response to a rhythmic incongrent condition for the target 2 + 2 rhythmic pattern was thought to reflect a detection of linguistic (grammatical) structure violation. After repeated exposure to primes, speakers constructed a rhythmic pattern consisting of the number of events and their corresponding temporal structure (different time intervals and global patterns). In addition, the musical-like rhythmic pattern and the grammatical structure of language were found to share a number of characteristics that may be intrinsically related^39^. Once the target speech was presented, speakers first parsed it into small parts (segmentation at the functional level), and then prepared their representation of the utterances’ prosodic structure^40,41^. Given that this N100 component was generated in an early stage during the read-aloud, it was plausible to assume that for the incongruent condition, this larger negative component could be linked to the detection of a hierarchical linguistic pattern that did not conform to the rhythmic structure of prime. This reflected that the segmentation for visually presented targets could be processed rapidly (e.g., at around 100 ms post-stimulus).

Studies have demonstrated the positive influence of rhythmically regular musical stimulation on grammatical task performance in German^42,43^, English^39^, and French^44–46^ native speakers. By contrast, the N100 component that Cason and Schön^8^ reported in response to a metrical mismatch was thought to reflect a violation of rhythmical expectation in a phoneme detection task. This was a speech perception task, and participants were asked to detect a target phoneme in auditorily-presented pseudowords, in which one first processed its rhythmic pattern and then detected a specified phoneme. Although the meaning of the component depended on the specific language processing, the facts that were consistent with the condition were processed more rapidly, which supported the findings that attentional orienting facilitated speech production or perception similarly. The N100 component here seemed like a mismatched negativity (MMN) response elicited by the primary auditory cortex, which was elicited by a rare deviant sound within a series of repetitive standard sounds^47–49^. The MMN is thought to reflect the brain’s ability when comparing auditory information against the short-term memory trace of auditory features. The MMN might also relate to a more general mechanism of expectancy violation. The MMN has been shown to reflect violations of complex, metrical pattern^50^, or sequential pattern^51,52^.

For the 2 + 2 target rhythm, the incongruent condition elicited a larger negative component than the congruent condition in the 350- to 500-ms range did, and this congruent effect was distributed bilaterally (see map distribution in Fig. 2).

We suggested that this effect indicated a violation of the rhythmic expectation during prosodic encoding in speech production. During the planning stages of prosodic information, it was probably more relevant to rapidly prepare each syllable and to generate a precise internal rhythmic representation of temporal structure^41^. In the current study, although the duration of each syllable (about 300 ms) in the target speech was distinct from that of each tone (100 ms) in local primes, the rhythmic pattern was congruent or incongruent between primes and targets globally. In other words, primes elicited an abstract rhythmic structure, which provided a prosodic frame, and they facilitated production of target speeches. We thus hypothesised that before target speech articulation, speakers generate a metrical frame during prosodic encoding, as predicted by the speech production model proposed by Levelt *et al*.^40^. This finding also suggested that the processing mechanisms of rhythmic patterns in speech production and perception in general are similar.

The difference between the congruent and incongruent rhythmic pattern was observed in the 350- to 500-ms N400 component, which was associated with lexical information processing and contextual integration in language comprehension (see Federmeier^53^ for a review). It has been demonstrated that the N400 amplitude is modulated by expectancy^54^, which reflected the level of integration difficulty of a word in a given sentence context^55,56^. However, the exact mechanism of the N400 was unclear so far. Rothermich *et al*.^12^ found that semantically unexpected words elicited an N400 that was significantly smaller in a metrically regular than a metrically irregular sentence context, which supported the view that metrical regularity enhances the prediction of phonological processing (i.e., stress location) in sentence comprehension. Furthermore, Magne *et al*.^57^ reported that semantically congruous but metrically incongruous words produced the highest error rates in a semantic task. In other words, participants usually judged the metrically incongruous words as semantically incongruous even when they were appropriate to the semantics. These findings indicated that, in general, N400 reflected an incongruous state in one’s cognitive system^58^. Considering that reading speech aloud involves speech segmentation and prosodic planning before articulation, it is plausible to assume that the N400 reflected the detection of incongruent rhythmic pattern between primes and targets at the stage of prosodic encoding in speech production.

With regard to the underlying mechanism, both components might have related to dynamic attention or neural entrainments. A substantial body of literature has suggested that the brain responds rhythmically to external rhythms^59,60^, and that this rhythmic activity influences the detection performance for a number of cycles after the offset of the external rhythms. Rohenkohl *et al*. indicated that rhythmic expectations were automatically formed^61^. In a MEG study, Oever *et al*.^62^ observed that neural phase-locking for rhythmic sounds occurred at intensities substantially below the detection thresholds, which indicated that neural entrainment was responsible for enhanced auditory sensitivity to rhythmic sounds, even for sub-threshold stimuli. The basis of neural entrainment is possibly automatic and stimulus-driven, which needs to be investigated further^63^.

Alternatively, it is possible that the N400 is related to speech motor synchronization in speech production. Studies have suggested that auditory rhythmic patterns may have an effect on motor entrainment, and that an external auditory stimulus can facilitate improved speech motor entrainment in children and adults while repeating the syllable as compared with motor entrainment when no external auditory stimulus was supplied. That is to say, external auditory stimulus can positively influence speech motor synchronization^64^. Reading latencies were around 670 ms, and speakers had the potential to programme phonetic scripts before articulation^40,65^. This suggested that the later component (N400) might also relate to speech motor synchronization. By contrast, a similar component observed in language comprehension (or detection) cannot reveal this possibility. Further study needs to be done to entangle these two possibilities (neural entrainment or speech motor synchronization) for the N400 component by comparing tasks with and without speech motor requirement.

The priming effect in the 1 + 3 rhythmic prime’s condition was absent in the present study, which suggested that speakers were not sensitive to the changes of this kind of rhythmic patterns. As mentioned in the introduction, the rhythmic pattern 1 + 3 is irregular for a four-character phrase’s utterance. In other words, speakers generate more 2 + 2 rhythmic patterns than 1 + 3 rhythmic patterns, which may form distinct sensitivity for different rhythmic patterns. For the target speeches with a regular rhythmic pattern, speakers can detect the inconsistency between primes and targets easily. While for the target speeches with an irregular rhythmic pattern, speakers probably were not sensitive the inconsistency between primes and targets. We thus did not observe this weak effect in the present study. Consistently with our speculation, Luo and Zhou^17^ reported a larger difference on the mean amplitude for the normal rhythmic patterns, while a smaller difference for the abnormal rhythmic patterns, during Chinese sentence reading task. These findings suggest that the frequency of rhythmic abstract pattern has an impact on the speech perception and production.

At the electrophysiological level, because of the difference on frequency between regular and irregular rhythmic patterns, it was probably more difficult to form neural or motor synchronization for the 1 + 3 rhythmic pattern in comparison with the 2 + 2 rhythmic pattern. This theory needs to be examined further by increasing the repeated times of the primes. Additionally, there was a difference in syntactic structure between the target 2 + 2 and 1 + 3 rhythmic pattern, which might exert the influence on the prosodic encoding in speech production due to the bidirectional activation between syntax and prosody^66^. Studies in Indo-European languages have demonstrated that linguistic structure building can clearly benefit from prosodic^67,68^, statistical cues^69^, or purely based on speakers’ grammatical knowledge. Further investigation needs to disentangle these factors.

In summary, the behavioural and electrophysiological findings of our study indicated a rhythmic priming effect in speech production. An early component (N100) elicited in response to target speeches that were rhythmically mismatched to primes was be linked to the detection of hierarchical linguistic units that did not conform to expectation, whereas a later negative component (N400) was thought to reflect the violation in the expectation of rhythmic pattern in speech production. Both components could have been related to dynamic attention or neural entrainments. Furthermore, the N400 component could have also related to speech motor synchronization at the stage of phonetic programming before articulation. To our knowledge, our findings provided the first electrophysiological evidence for the rhythmic priming effect in producing Mandarin spoken words. These findings suggest that rhythmic pattern constrains grammatical and prosodic encoding during speech production, and support the hypothesis that speakers form a grammatical or a prosodic abstract frame before articulation.

## Supplementary information

Experimental materials
