# Supplementary material for: Rhythmic pattern facilitates speech production: An ERP study
Source: Sci Rep. 2019 Sep 10;9:12974. doi: 10.1038/s41598-019-49375-8 (PMC6736834; doi:10.1038/s41598-019-49375-8)
Supplement: Supplementary file 1 — Experimental materials [file 41598_2019_49375_MOESM1_ESM.zip › Target phrases and sentences of 2+2 and 1+3 rhythmic pattern.docx]

Rhythmic pattern facilitates speech production: An ERP study

Ning Zhang, Qingfang Zhang

Target phrases and sentences of 2+2 and 1+3 rhythmic pattern

| 2+2 | 1+3 |
| --- | --- |
| 阿姨买菜 | 办信用卡 |
| 杯子破裂 | 背三字经 |
| 比赛开始 | 补维生素 |
| 冰雪融化 | 拆包装盒 |
| 博士毕业 | 炒西葫芦 |
| 超市关门 | 乘出租车 |
| 城市规划 | 吃闭门羹 |
| 大叔抽烟 | 吃冰激凌 |
| 大雁南飞 | 穿羊毛衫 |
| 歹徒抢劫 | 吹耳边风 |
| 单位放假 | 挡紫外线 |
| 地球自转 | 丢学生证 |
| 电脑中毒 | 读电子书 |
| 电视播放 | 发劳务费 |
| 飞机降落 | 付加班费 |
| 钢琴演奏 | 逛博物馆 |
| 公司破产 | 过斑马线 |
| 故事发生 | 过圣诞节 |
| 观众鼓掌 | 毁人生观 |
| 孩子认错 | 寄明信片 |
| 护士打针 | 讲真心话 |
| 花朵枯萎 | 嚼口香糖 |
| 活动结束 | 叫服务员 |
| 火车行驶 | 解方程组 |
| 角色扮演 | 进教学楼 |
| 距离缩短 | 开电风扇 |
| 科学进步 | 看电视剧 |
| 理想破灭 | 看说明书 |
| 毛衣缩水 | 考公务员 |
| 矛盾激化 | 列黑名单 |
| 皮肤过敏 | 拎塑料袋 |
| 屏幕显示 | 领补助金 |
| 钱包丢失 | 买化妆品 |
| 商品下架 | 念紧箍咒 |
| 食物变质 | 拍纪录片 |
| 寿命延长 | 泡图书馆 |
| 书店倒闭 | 喷杀虫剂 |
| 水管堵塞 | 骑电瓶车 |
| 速度放慢 | 切土豆丝 |
| 万物复苏 | 请临时工 |
| 网络断开 | 求平均数 |
| 温度上升 | 入共产党 |
| 文化传承 | 洒消毒剂 |
| 问题提出 | 扫办公室 |
| 相机拍照 | 数倒计时 |
| 心跳停止 | 说口头禅 |
| 雪花飘落 | 撕保鲜膜 |
| 阳光照耀 | 送土特产 |
| 药品过期 | 谈女朋友 |
| 业绩提升 | 选必修课 |
| 医生治病 | 学计算机 |
| 遗产继承 | 用笔记本 |
| 音乐鉴赏 | 有安全感 |
| 鹦鹉学舌 | 扎马尾辫 |
| 邮局送信 | 招本科生 |
| 游客爬山 | 找突破口 |
| 员工加班 | 致欢迎词 |
| 植物生长 | 煮方便面 |
| 指纹匹配 | 租写字楼 |
| 钻石闪耀 | 做红烧肉 |
